# Retrospective Case Series of Fulminant Metabolic Crisis in GSDIA: Persistent Lactic Acidosis Despite Correction of Hypoglycemia May Reflect Secondary Mitochondrial Dysfunction

**DOI:** 10.1002/jmd2.70059

**Published:** 2025-12-25

**Authors:** Herodes Guzman, Nicole Stewart, Lauren Mitteer, Victoria Sanders, Rebecca Ganetzky, Diva D. De Leon

**Affiliations:** ^1^ Division of Endocrinology and Diabetes Children's Hospital of Philadelphia Philadelphia Pennsylvania USA; ^2^ Division of Genetics Children's Hospital of Philadelphia Philadelphia Pennsylvania USA; ^3^ Department of Pediatrics Perelman School of Medicine at the University of Pennsylvania Philadelphia Pennsylvania USA

**Keywords:** GSD, hyperglycemia, lactic acidosis, mitochondria, mortality, thiamine

## Abstract

Although several advances in glycogen storage disorder (GSD) management have been made over the years, there is still increased morbidity and mortality in these patients, particularly among those with GSD type I (GSDI). Here, we describe a case series of five patients with GSDIa who presented with severe lactic acidosis and passed away without a clearly identified etiology, despite some having adequate metabolic control at baseline. Based on these presentations and emerging literature, we posit that progressive, secondary mitochondrial dysfunction plays an important role in placing GSDIa patients at increased risk for GSDI‐related complications, including fulminant metabolic crisis. Both *G6PC1* knockout mice and human GSDIa plasma and urinary samples exhibit markers of mitochondrial dysfunction. Our observations of refractory lactic acidosis despite hyper‐ or eu‐glycemia potentially suggest mitochondrial dysfunction as a unique feature of the metabolic decompensation experienced by the patients of this report. Thus, underlying mitochondrial dysfunction may set up certain GSDIa patients for multiorgan failure that is unresponsive to conventional therapies. Further research into the mitochondrial function of GSDIa patients across the lifespan is warranted. By introducing surveillance strategies for mitochondrial dysfunction in GSDI, existing antioxidant therapies could be harnessed to maintain or improve mitochondrial function in this patient population. In turn, the integration of mitochondrial therapies into GSD care may improve quality of life and avoid the development of fulminant metabolic crisis.

## Synopsis

Secondary mitochondrial dysfunction may place certain patients with glycogen storage disorder type Ia at increased risk for fulminant metabolic crisis despite adequate metabolic control.

## 1 Introduction

Glycogen storage disorder type Ia (GSDIa, OMIM #232200) is an autosomal recessive disorder of glucose homeostasis caused by pathogenic variants in the glucose‐6 phosphatase catalytic subunit 1 (*G6PC1*) gene [1]. Resulting deficiency of glucose‐6‐phosphatase disrupts both glycogenolysis and gluconeogenesis [1]. Affected individuals typically present at 3–6 months of age with hypoglycemia, lactic acidosis, hypertriglyceridemia, hyperuricemia, short stature, hepatomegaly, and nephromegaly [1, 2]. Current management is supportive with a focus on limiting fasting time through frequent complex carbohydrate meals and repeated administration of uncooked cornstarch [1, 2]. Acute episodes of metabolic decompensation, triggered by illness and/or fasting, are characterized by severe hypoglycemia with lactic acidosis. Hypoglycemia in GSDIa induces lactic acidosis by engaging gluconeogenesis to compensate for defective glycogenolysis and glucose release from the liver [3]. With a block in conversion from glucose‐6‐phosphate to glucose, glucose‐6‐phosphate is shunted to alternative metabolic pathways, including conversion of pyruvate to lactate. A build‐up of lactate with hypoglycemia can provoke lactic acidosis but is often reversible by achieving euglycemia and shutting off gluconeogenesis [3]. Despite optimal metabolic management, these patients remain at risk for metabolic crises and long‐term complications, including nephropathy, nephrolithiasis, metabolic bone disease, and hepatic adenomas or hepatocellular carcinoma, among other complications [1, 2]. Novel therapeutics to address disease burden are clearly needed and clinical trials for gene replacement therapy are ongoing [2, 4, 5].

Emerging research suggests that secondary mitochondrial dysfunction plays an important role in GSDI pathophysiology. Altered glycogen metabolism is thought to impair mitochondrial function through various mechanisms, including limiting glucose availability for oxidative phosphorylation [6]. Reduced mitochondrial content, altered mitochondrial structure, impaired aerobic respiration, and dysfunction of the tricarboxylic acid (TCA) cycle have all been observed in mouse models of GSDIa [6]. Moreover, mitophagy is interrupted, resulting in an accumulation of damaged mitochondria [6]. With progressive mitochondrial dysfunction, oxidative stress increases and cellular injury ensues. Over an individual's lifespan, such damage may contribute to the development and progression of GSD‐related complications and leave some individuals vulnerable to fulminant metabolic crises in the setting of illness or physical stress.

Recent findings of secondary mitochondrial dysfunction reveal an opportunity to optimize GSD care through placing greater focus on mitochondrial health. In this retrospective case series, we present available data on five cases of GSDIa who presented with unexplained fulminant metabolic crisis and died after refractory lactic acidosis despite receiving maximal standard therapies. We posit that underlying mitochondrial dysfunction may have played a part in their clinical course and ultimate outcome.

## 2 Case Reports

We collected the following clinical data from our GSD cohort through retrospective chart review. Both electronic medical records and paper charts were reviewed to characterize each case in detail. We provide an assessment of each patient's baseline metabolic health status using medical documentation available immediately before their final hospitalization. Metabolic health status was informed by provider documentation and available metabolic screening labs, including liver transaminases, lipid panels, and uric acid level, among others. The identified cases represent our institutional experience with GSDIa outcomes over the past 18 years and are presented in chronological order according to the year of their deaths.

### 2.1 Case 1

The first patient was a 14‐year‐old White female who was first diagnosed with GSDIa at 3 months of age due to hepatomegaly incidentally noted during a hospitalization for febrile illness with hypoglycemia and lactatemia. A liver biopsy demonstrated 5.9% glycogen as % weight of wet tissue (reference range [RR]: 2.5–6.0) with glucose‐6‐phosphatase activity of 0.114 um phosphate/g/min (RR: 4.7 ± 1.9), which confirmed her diagnosis of GSDIa. She was diagnosed before genetic testing was available commercially, thus, genetic diagnosis was not completed. At baseline, this patient had nephrolithiasis and co‐morbidities of mild intermittent asthma, migraines, depression with suicidal ideation, and absence seizures. She was described to be adherent to her GSDIa treatment regimen and in good metabolic control, though documentation of GSD surveillance lab work were not available in her medical records during the year of her death. She was managed with uncooked cornstarch every 6 h (total daily dose [TDD]: 4.6 g/kg).

Patient 1 presented to an outside hospital with 24 h of “not feeling well” with “whole body” pain and two episodes of small‐volume emesis. Prior to admission, the family attempted to manage her symptoms at home with Powerade every 2 h and a mix of Polycose, Prosobee formula, and cornstarch. On the day of presentation, she became progressively more lethargic and refused to walk due to weakness. Enroute to the hospital, her initial plasma glucose (PG) demonstrated euglycemia at 140 mg/dL. Upon arrival, her physical exam was notable for tacky mucous membranes, rapid breathing, and sinus tachycardia. Initial labs at our hospital demonstrated hyperglycemia, severe anion gap metabolic acidosis, hepatic dysfunction, acute kidney injury, and pancreatitis with PG 207 mg/dL, pH 6.80, bicarbonate (HCO3) < 5 mmol/L (RR: 20–26 mmol/L), alanine aminotransferase 149 U/L (RR: 5–30 U/L), aspartate aminotransferase 545 U/L (RR: 10–30 U/L), creatinine 2.5 mg/dL (RR: 0.4–1.0 mg/dL), and lipase 2064 U/L (25–110). She was started on 10% dextrose fluids at a maintenance rate, which was increased to 1.5 times her maintenance rate at transfer to our institution. Here she rapidly progressed to respiratory failure with labs reflecting ongoing metabolic acidosis (pH 6.78 and HCO3 3.5 mmol/L on venous blood gas). She subsequently developed multiorgan failure with severe hypotension and oliguria. Due to a rapidly increasing abdominal girth she was emergently taken to the operating room for an exploratory laparotomy where no bowel ischemia was identified macroscopically, though she was noted to have chronic appendicitis. Her appendix was removed, and she was returned to the pediatric intensive care unit (PICU). There, vasopressor support was increased throughout the night until she experienced cardiac arrest. Despite receiving cardiopulmonary resuscitation (CPR) for 40 min, return of spontaneous circulation (ROSC) was not achieved. Throughout the resuscitative efforts, she remained hyperglycemic with PG above 300 mg/dL before achieving euglycemia on hospital day 2, which was maintained with final recorded PG 124 mg/dL. Despite PG normalization, her lactate progressively increased from 16.1 to > 27 mmol/L (RR: 0.5–1.6) and remained above the limit of detection before time of death. All infectious cultures returned negative. A toxicology screen was also unrevealing. No autopsy was performed. Of note, the patient was hospitalized a month prior to her presentation. During that hospital stay, she was treated for crampy abdominal pain and emesis with hypoglycemia and lactic acidosis that was reversed after 2 days of 10% dextrose fluid administration at a maintenance rate.

### 2.2 Cases 2 and 3

Patients 2 and 3 are siblings and each of their final presenting hospitalizations occurred 4 years apart. The second patient was a 3‐year‐old Black female who was diagnosed with GSDIa at birth due to a family history of the disease in her older sibling (patient 3). Targeted panel testing revealed her to carry the familial c.79delC (p.Q27Rfs*9) *G6PC1* variant and reportedly a novel p.Y323Ter (c.1048C>A) variant, confirming her diagnosis. At baseline, patient 2 was developmentally appropriate, overweight, and had hepatomegaly. For her GSDIa, she was treated with every 3–5‐h uncooked cornstarch (TDD: 9.1 g/kg) administered through a gastrostomy tube. Metabolic labs 4 months prior to admission reflected good metabolic control with total cholesterol 142 mg/dL (RR: 45–182 mg/dL), triglycerides 62 mg/dL (RR: 27–125 mg/dL), high‐density lipoprotein 59 mg/dL (RR: 35–82 mg/dL), low‐density lipoprotein 71 mg/dL (RR < 110 mg/dL), uric acid 4.0 mg/dL (RR: 1.8–5.0 mg/dL), c‐reactive protein < 0.5 mg/dL (RR < 1.0 mg/dL), sedimentation rate 9 mm/h (RR: 0–20 mm/h), hemoglobin 12.3 g/dL (RR: 11.5–13.5 g/dL), alanine aminotransferase 13 U/L (RR: 5–45 U/L), and aspartate aminotransferase 44 U/L (RR: 20–60 U/L).

After an uneventful night, patient 2 awoke the morning of presentation with decreased energy and not acting like herself. Upon arrival to our institution, she was immediately taken to the resuscitation bay for hypothermia, bradycardia, and hypotension. CPR was started for cardiac arrest, and she was taken to the PICU intubated onto mechanical ventilation. Initial labs revealed acute kidney injury (creatinine 1.9 mg/dL [RR: 0.1–0.4 mg/dL]) with a severe anion gap metabolic acidosis with pH 6.93, HCO_3_ < 5 mmol/L, and lactate 24.1 mmol/L. The first available PG around the time of her arrival was 87 mg/dL. Hepatic function was not significantly deranged initially with alanine aminotransferase 36 U/L (RR: 5–45 U/L) and aspartate aminotransferase 140 U/L (RR: 20–60 U/L). Multiple vasopressors, 100% fraction of inspired oxygen, and inhaled nitric oxide were added. Although patient 2 did not have a known history of hypothyroidism, thyroid hormone infusion was started to address thyroid screening labs that were most consistent with non‐thyroidal illness (thyroid‐stimulating hormone 1.30 mIU/mL, free thyroxine 0.7 ng/dL, and triiodothyronine < 0.4 ng/mL) [7]. No documentation is available to confirm dextrose fluid administration. She remained in pressor refractory shock, quickly became anuric, developed abdominal compartment syndrome, and progressed to multiorgan failure. After multiple family meetings, the family elected to withdraw care. Aside from a short period of hyperglycemia to a peak of 198 mg/dL, patient 2 maintained euglycemia throughout with all PGs > 70 mg/dL. Despite correction of acidosis, lactates remained above the limit of detection at > 27 mmol/L. All infectious cultures returned negative. An autopsy did not reveal conclusive evidence of her cause of death—diffuse bilateral bronchopneumonia with alveolar damage was appreciated but with negative postmortem bacterial and fungal cultures and negative viral studies. A degree of brain swelling was also noted. No biopsies were taken from her liver or kidneys for organelle‐specific evaluation.

Notably, patient 2 was discharged from our institution 2 days prior to re‐presenting at the emergency department as described above. She had been admitted to the hospital for 2 weeks for new‐onset, non‐hypoglycemic seizures of unclear etiology requiring levetiracetam therapy. During this time, she experienced seven distinct episodes of seizure activity characterized by eye rolling and blinking, arm shaking and stiffness, hand clenching, and urinary and fecal incontinence. Initial lactic acidosis with lactate of 18 mmol/L and bicarbonate of 8 mmol/L resolved with 10% dextrose fluid administration at 1.5 times her maintenance rate. She experienced repeated hyperglycemia to the 200's mg/dL throughout this hospitalization and did not have documented episodes of hypoglycemia. Both magnetic resonance imaging of the brain and electroencephalogram returned normal. Urine toxicology was negative. No infectious trigger was identified. She required 700 mg twice daily levetiracetam maintenance dosing to control seizure activity.

Patient 3 was a 9‐year‐old Black female who was diagnosed with GSDIa at 3 months of age after being hospitalized for black‐colored emesis and watery stools likely resulting from viral illness. During this stay, she was appreciated to have hepatosplenomegaly and elevated liver transaminases. A liver biopsy completed that admission confirmed her GSDIa diagnosis. Although targeted panel testing only revealed one familial c.79delC (p.Q27Rfs*9) *G6PC1* variant, comprehensive genetic testing was not readily available to confirm additional variants. At baseline, patient 3 was developmentally appropriate with hepatomegaly, bilateral nephrolithiasis, and a gastrostomy tube for nutrition and uncooked cornstarch. She was reported to be in fair metabolic control with every 4‐h uncooked cornstarch (TDD: 6.3 g/kg) and two overnight lactose‐free formula feeds.

On the day of presentation to our institution, patient 3 had been experiencing acute onset stomach pain, diarrhea and repeated emesis with gastrostomy tube feed administration over the past 24 h after being discharged home from a previous hospitalization with our team. She had been electively admitted to the hospital for 4 days to trial bedtime Glycosade (extended‐release uncooked cornstarch). Given that Glycosade did not extend her overnight fasting tolerance, this was not continued, and she was maintained on her regular cornstarch regimen at discharge from this hospital stay. Upon arrival to the emergency room with her current gastrointestinal symptoms, she was found to be obtunded and cool to the touch with initial PG 19 mg/dL, pH 6.709, HCO_3_ 4.5 mmol/L, lactate > 27 mmol/L, blood urea nitrogen 32 mg/dL (RR: 5–17 mg/dL), creatinine 3.4 mg/dL (RR: 0.2–0.5 mg/dL), sodium 140 mmol/L (RR: 136–142 mmol/L), potassium 6.9 mmol/L (RR: 3.8–5.0 mmol/L), ionized calcium 1.25 mmol/L (RR: 1.15–1.34 mmol/L), alanine aminotransferase 1321 U/L (RR: 10–35 U/L), aspartate aminotransferase 3746 U/L (RR: 15–40 U/L), and ammonia 251.6 umol/L (RR: 9–33 umol/L). After her PG was raised to 65 mg/dL with an enteral 50% dextrose bolus, she was taken to the resuscitation bay. There, she went into asystole and CPR was initiated. After return of spontaneous circulation was achieved, she was intubated, placed on 10% dextrose fluids without potassium at ~1.5× her maintenance rate, dopamine and epinephrine infusions, provided calcium gluconate for developing hypocalcemia and empiric antibiotics for possible underlying infection, and given multiple sodium bicarbonate boluses to correct her acidosis. An echocardiogram taken ~3 h prior to inotrope initiation revealed normal cardiac function. General Surgery was consulted to determine whether abdominal pathology could explain patient 3's presentation. An abdominal x‐ray did not reveal an underlying abdominal process and their exam was benign. Given anuric renal failure, refractory metabolic acidosis, and hyperkalemia, continuous renal replacement therapy (CRRT) was initiated. Although her acidosis was corrected with ongoing sodium acetate infusion, her lactates remained high at > 27 mmol/L. She went on to develop pressor refractory hypotension the night of her presentation and passed away after experiencing repeat cardiac arrest. Along with continuous 10% dextrose fluid administration, she was started on an insulin infusion to treat persistent hyperglycemia that developed quickly after presentation. Although she received stress‐dose hydrocortisone several hours into her stay, she became hyperglycemic to the 300's mg/dL beforehand. An autopsy revealed bilateral bronchopneumonia with patchy pulmonary hemorrhage suggestive of an infectious process; however, all infectious cultures returned negative. No biopsies were taken from her liver or kidneys for organelle‐specific evaluation.

### 2.3 Case 4

Patient 4 was a 31‐year‐old White male who was diagnosed with GSDIa at 4 months of age after presenting to care with growth faltering, hepatomegaly, and hypertriglyceridemia. At diagnosis, he was found to have post‐prandial hypoglycemia and was not responsive to glucagon. As such, he was diagnosed with GSDIa based on clinical features. He did not have molecular confirmation of GSDIa due to limitations in genetic testing at the time of his diagnosis. After a period of no follow‐up, he re‐established care with our institution at 29 years old. At that time, he reported having early morning hypoglycemia 2–3 times per week while taking 14 g dextrose powder combined with uncooked cornstarch every 5–6‐h (TDD of cornstarch: 2.9 g/kg). He was not adherent to his cornstarch regimen and often fasted 10 h overnight. In addition to hepatomegaly and hypertriglyceridemia, he also suffered from elevated liver transaminases, hyperuricemia, recurrent nephrolithiasis, nephrocalcinosis, and co‐morbidities of chronic headache and recurrent diarrhea. He reported smoking one pack per day of cigarettes.

About a week prior to presentation, patient 4 described feeling unwell or “hungover” 2 days after drinking alcohol recreationally. He was having intermittent emesis and started experiencing slight confusion the day prior to presentation. PG was reportedly normal with one documented PG 146 mg/dL. The night of his presentation, he was complaining of vague, left‐sided abdominal pain, blurry vision, and hyperglycemia to the 400's mg/dL, and became increasingly confused. Upon arrival to an outside hospital, he was tachypneic and confused with very dry mucous membranes. He was found to be in hypoxemic respiratory failure and was intubated onto mechanical ventilation. Initial labs revealed hyperglycemia and severe lactic acidosis with PG 509 mg/dL, lactate > 20 mEq/L (RR: 0.5–2.2 mEq/L), HCO3 < 5 mEq/L (RR: 24.0–31.0 mEq/L), and trace urine ketones. A hemoglobin A1c of 4.9% with hemoglobin of 13.9 g/dL (RR: 14.0–17.5 g/dL) ruled out new‐onset diabetes mellitus. Acute kidney injury and hepatic dysfunction were present with creatinine 2.76 mg/dL (RR: 0.50–1.50 mg/dL) and alanine aminotransferase of 65 IU/L (RR: 2–38 IU/L) with aspartate aminotransferase of 190 IU/L (RR: 9–37 IU/L). All infectious cultures were negative. After intubation, he became bradycardic and went into asystole, achieving ROSC after epinephrine and sodium bicarbonate administration. He had recurrent cardiac arrest, once from a witnessed aspiration event, requiring escalation of vasopressor support. He was transferred to the medical ICU where pulmonary imaging demonstrated bilateral basilar infiltrates consistent with aspiration and probable acute respiratory distress syndrome. Abdominal imaging showed findings consistent with ischemic bowel. Head CT revealed signs of accelerated aging but no acute brain injury. For his bowel findings, he went for an emergent exploratory laparotomy with no signs of ischemic colitis, just generalized hypoperfusion. Upon return from the operating room, he arrested again and developed multisystem organ failure. He passed away before hemodialysis or extracorporeal membrane oxygenation (ECMO) could be initiated. No autopsy was performed. Throughout his hospitalization, his PG ranged in the 200–300's mg/dL and his lactates were persistently > 20 mEq/L. His acidosis was only partially corrected to a bicarbonate of 8.2 mEq/L.

### 2.4 Case 5

Patient 5 was a 17‐year‐old White male diagnosed with GSDIa at birth due to a family history of the disease in his older sibling. Targeted panel testing revealed compound heterozygous pathogenic variants in *G6PC1* (c.247C>T, p.Arg83Cys; c.79delC, p.Gln27Argfs*9). At baseline, patient 5 was developmentally appropriate, obese, had hepatomegaly, nephromegaly, hypertension, hyperuricemia, nephrolithiasis, proximal tubular dysfunction, hypocitraturia, recurrent pancreatitis, vitamin D deficiency, anxiety, depression, and self‐injurious behavior. He was not completely adherent to his every 5–6‐h daytime uncooked cornstarch regimen and 150 g Glycosade at bedtime (TDD of cornstarch: ~2.8 g/kg). He was started on a Dexcom G6 continuous glucose monitor a year prior to his final hospitalization.

Shortly after being discharged home from the hospital for repeated emesis due to a viral infection, patient 5 reported developing worsening fatigue, shortness of breath, tachycardia, and bilateral leg discomfort and swelling 3–4 weeks prior to presentation. Due to his progressive symptoms, he initially presented to an outside hospital where he was found to have an elevated troponin and normal function on echocardiogram. His lactate was slightly elevated to 7 mmol/L (RR: 0.5–1.6 mmol/L), up from his baseline of 2–4 mmol/L.

He was transferred to our institution with concerns for myocarditis. Upon arrival, he was tachypneic, tachycardic, hypertensive, and tired appearing with bilateral leg swelling and significant weight gain of 4.3 kg over the course of a month. Repeat echocardiogram continued to show normal biventricular function, though with incidental finding of secundum atrial septal defect, and beta‐type natriuretic peptide was normal at 76.5 pg/mL (RR: 0.0–100.0 pg/mL), so concern for myocarditis decreased. Initial labs showed lactic acidosis with pH 7.38, bicarbonate 10 mmol/L, lactate 11.6 mmol/L, and PG 74 mg/dL. All infectious cultures returned negative. After he was confirmed to have normal cardiac function, his fluids were increased to 12.5% dextrose run at 1.5 times his maintenance rate. Multiple doses of furosemide were administered to alleviate peripheral edema. Over the course of 3 days in the inpatient unit, he was unable to be weaned off dextrose fluids due to repeated emesis with oral intake.

Given findings of pulmonary hypertension on repeat echocardiogram, he was started on inhaled nitric oxide and transferred to our cardiac ICU. Over the course of 2 days, his lactate spiked to > 25 mmol/L with pH 6.927 and bicarbonate 2.3 mmol/L despite maintenance of euglycemia. He developed acute kidney injury and worsening hepatic function around the same time with creatinine rising to 1.33 mg/dL (RR: 0.30–0.80 mg/dL) and alanine aminotransferase of 79 U/L (RR: 10–35 U/L) with aspartate aminotransferase of 313 U/L (RR: 15–45 U/L). He became tachypneic and encephalopathic, with hematochezia appreciated and suspected to be due to a low‐flow state. He subsequently went into ventricular fibrillation arrest, requiring several hours of CPR. He received multiple sodium bicarbonate boluses and was transitioned to an infusion. Multiple vasopressors, stress dose hydrocortisone, total parenteral nutrition, and an insulin infusion were also started. The following day, CRRT was initiated to help clear his lactic acidosis, but he developed severe pulmonary edema with line placement and had to be emergently intubated. Although the acidosis improved, his lactate remained high at > 25 mmol/L. He was placed on veno‐arterial ECMO later that day. Given severe vasoplegia, methylene blue was initiated the next day. High‐dose intravenous thiamine was also started under the direction of the biochemical genetics team because of a known association with critical illness and thiamine deficiency causing secondary impairment of the pyruvate dehydrogenase complex and lactic acidosis. This was supported by elevation of 2OH‐isovaleric acid in the urine and a small amount of alloisoleucine in the plasma. Additionally, he was found to have an elevated ratio of lactate to pyruvate of 30 (RR: 10–20) in the blood. Notably, this was his first known exposure to thiamine supplementation. Comprehensive genetic testing, including mitochondrial DNA sequencing, was sent but returned only showing the known *G6PC1* variants. He awoke later that day, and his lactate was successfully decreased to 4–5 mmol/L. Over the course of 2 days, he passed away from progressive multisystem organ failure and repeated cardiac arrest despite maintenance of euglycemia, normalized lactates and resolved acidosis. An autopsy did not find a definitive cause of death, though heart and lungs were not available due to organ donation. Postmortem bacterial and fungal cultures were negative. Acute pancreatitis with areas of necrosis was appreciated and likely contributed to his hyperglycemia later in his course based on collected pancreatic markers (amylase 37 U/L [RR: 30–100 U/L] on hospital day 4 of 10 and 249 U/L on hospital day 8 while lipase 47 U/L [RR: 23–300 U/L] on hospital day 4 and 1299 U/L on hospital day 8). No biopsies were taken from his liver or kidneys for organelle‐specific evaluation.

**FIGURE 1:**
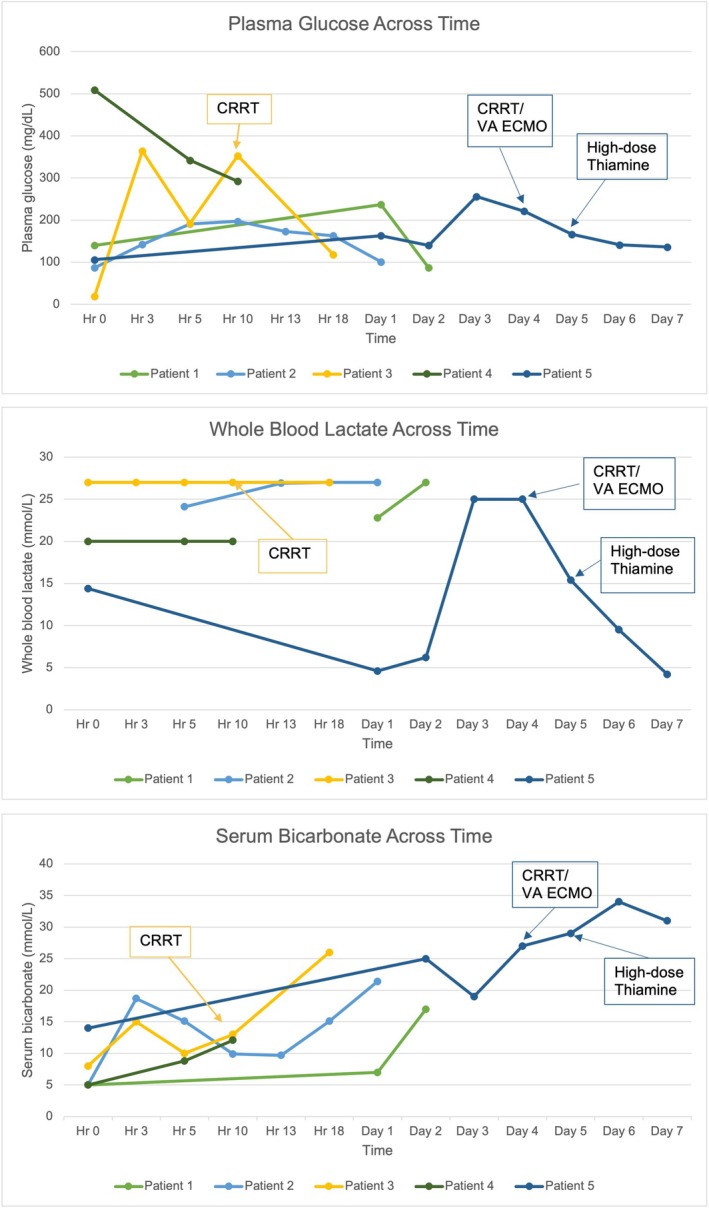
Plasma glucose, whole blood lactate, and serum bicarbonate across time for GSDIa patients during fulminant metabolic crisis. *CRRT: Continuous renal replacement therapy; Hr: Hour; VA ECMO: Veno‐arterial extracorporeal membrane oxygenation. Patients who were placed on CRRT and VA ECMO are highlighted for comparison.

A clinical summary of these cases is provided in Table 1 with detailed laboratory studies collected during each final hospital encounter available in Table S1. Available glucose, lactate, and bicarbonate trends during the final hospitalization of each patient is shown in Figure 1.

**TABLE 1:** Clinical characteristics of GSDIa patients at baseline and at time of fulminant metabolic crisis.

Case	Sex	Age^b^ (years)	Diagnosis method	Genotype	GSD management	Co‐morbid conditions	Presenting symptoms	Initial labs	Repeat hosp. (Y/N)
1	F	14	Liver biopsy	Not available	Q6H cs (4.6 g/kg/day)	Asthma, migraines, depression, absence seizures, chronic appendicitis	Malaise, generalized pain, emesis	PG 140, lactate 16.1	Y
2^a^	F	3	Genotype, family history	c.79delC, c.1048C>A	Q3‐5H cs (9.1 g/kg/day)	Overweight	Somnolence	PG 135, lactate 24.1	Y
3^a^	F	9	Genotype, liver biopsy	c.79delC, —	Q4H cs (6.3 g/kg/day)	—	Emesis, diarrhea, obtundation	PG 19, lactate > 27	Y
4	M	31	Clinical	Not available	Q5‐6H 14 g dextrose powder with cs (2.9 g/kg/day)	Chronic headache, recurrent diarrhea, cigarette smoking	Confusion, blurry vision, emesis, abdominal pain	PG 509, lactate > 20	N
5	M	17	Genotype	c.247C>T, c.79delC	Q5‐6H daytime cs, QHS 150 g Glycosade (2.8 g/kg/day)	Obesity, recurrent pancreatitis anxiety, depression	Fatigue, dyspnea, tachycardia, bilateral leg discomfort	PG 74, lactate 11.6	Y

Abbreviations: cs: cornstarch; F: female; GSD: glycogen storage disease; hosp.: hospitalization; M: male; N: no; PG: plasma glucose; QHS: nightly; QXH: every X hour; Y: yes.

^a^ Siblings.

^b^ Age at presentation; gastrointestinal symptoms are bolded; PG (mg/dL), Lactate (mmol/L).

## 3 Discussion

Despite advances in the care of GSDIa, increased morbidity and mortality remain an issue for this patient population, including recurrent metabolic crises characterized by profound hypoglycemia and lactic acidosis [8]. Typically, restoration of euglycemia with dextrose fluids resolves lactic acidosis and has been concordant with our institution's experience with GSDIa [3, 9]. In this case series, we highlight patients identified over the past 18 years who presented with unexpectedly severe metabolic crisis and refractory lactic acidosis despite having hyperglycemia or achieving euglycemia. What we notice is a pattern of vague illness presentation without clear infectious etiology, persistent hyperglycemia with profound lactatemia, and the development of multisystem organ failure from refractory hypotension due to metabolic acidosis. We suspect that refractory hyperlactatemia in these cases could have resulted from both chronic and acute secondary mitochondrial dysfunction: progressive mitochondrial dysfunction over these patients' lifetime may have compromised baseline hepatorenal function, which then impaired clearance of excess lactate generated from glycolytic overcompensation during illness that is diverted by a dysfunctional TCA cycle and respiratory chain. Worsening hepatorenal dysfunction then likely propagated subsequent cardiac dysfunction and refractory hypotension [9]. Although this process is most likely multifactorial, secondary mitochondrial dysfunction could play a role in promoting this cascade of events. For one of our historical cases, we note alcohol consumption prior to his demise, which is a known mitochondrial toxin [10]. Other potential predictors of mortality we appreciated across patients include baseline gastrointestinal symptoms that precluded nutritional optimization and rehospitalization. More importantly, evidence available in each patient's medical record of adequate metabolic control did not prevent fulminant metabolic crisis.

Lactic acidosis arises from mitochondrial dysfunction because of impairment of the cellular redox state, resulting in insufficient nicotinamide adenine dinucleotide (NAD+). To regenerate cytosolic NAD+, pyruvate is reduced to lactate, rather than being oxidized, resulting in accumulation of lactate. TCA cycle function and oxidative phosphorylation are also essential for insulin release and cellular response to insulin; therefore, their impairment could explain the persistent hyperglycemia observed in our cases. It is known that patients with critical illness from a plethora of causes may experience thiamine deficiency and secondary impairment of the pyruvate dehydrogenase complex. This may explain the resolution of lactatemia in patient 5 who received CRRT and high‐dose thiamine supplementation when compared to patient 3 who only received CRRT [11, 12]. However, this is confounded by the receipt of veno‐arterial ECMO in patient 5. We suspect that patient 5 passed away despite successfully lowering his lactate because he had already experienced significant and irreversible organ damage by the time thiamine supplementation was started. While liver transplantation can be considered for GSDIa patients with inadequate metabolic control, it was not considered in any of our patients because of their presumed metabolic stability before their demise [13]. Although multiple factors likely resulted in the demise of the cases presented here, highlighting the potential role of mitochondrial dysfunction represents an opportunity for intervention through greater attention to mitochondrial health surveillance.

The persistent lactatemia we observed in each case of metabolic crisis is likely a combination of increased lactate production and decreased clearance. Although tissue hypoperfusion often explains lactate production in the critical care setting, mitochondrial dysfunction may contribute through stimulation of lactate dehydrogenase, which reduces pyruvate to lactate [6, 9]. Defective oxidative phosphorylation results in a decreased NAD+ to nicotinamide adenine dinucleotide plus hydrogen (NADH) ratio, stimulating lactate dehydrogenase to oxidize more NADH to NAD+ [6]. Increased equilibrium of the lactate dehydrogenase to favor lactate production has been reported in the peripheral lymphocytes of a small group of GSDIa patients when compared to healthy controls [14]. Glycolytic overcompensation during acute crisis likely drives further lactate production, which may worsen with continuous dextrose administration [6]. Even though we cannot conclude thiamine supplementation facilitated aerobic respiration and resolution of lactatemia in patient 5, high‐dose thiamine has been shown to improve lactic acidosis in pyruvate dehydrogenase deficiency, and possibly in other critically ill patients [11, 15]. Moreover, the lack of serious side effects makes thiamine supplementation an inviting adjunctive therapy to trial in GSDIa patients in fulminant metabolic crisis [16, 17, 18]. Other promising supportive therapies at our disposal include nicotinamide riboside and dichloroacetate. Extensive research has shown that nicotinamide riboside, an NAD+ precursor, reduces neuroinflammation, slows the aging process, and can be cardioprotective [19]. On the other hand, dichloroacetate, an activator of the pyruvate dehydrogenase complex, has been revealed to decrease lactic acidosis in a broad range of conditions, including sepsis, trauma, and mitochondrial disease [20]. Therapies that do not directly lower lactate but optimize mitochondrial function, including coenzyme Q10, should also be considered in acute metabolic crises [15]. However, future research into the efficacy of these therapeutics in fulminant metabolic crisis of GSDIa still needs to be conducted.

Current therapeutic strategies were unable to restore metabolic stability in our GSDIa patients. Short‐term mitochondrial‐directed therapy does not appear to reverse fulminant metabolic crisis either, though these therapeutics (i.e., thiamine) were only considered and trialed in one of our presented cases. Thus, attempts to understand the long‐term and possibly progressive role of mitochondrial dysfunction in GSDIa are essential. Prior research suggests that the biochemical perturbations observed in GSDI lead to gluco‐ and lipotoxicity that damage mitochondria over time [21, 22]. Deeper insights derived from *G6PC1* knockout mice show an association between GSDIa and decreased liver mitochondrial content, altered mitochondrial structure, dysfunctional respiration, and aberrant TCA cycle function [6, 13]. Furthermore, *G6PC1* knockout mice experience downregulation of hepatic sirtuin 1 signaling, which decreases peroxisome proliferator‐activated receptor gamma coactivator‐1 alpha activity and leads to impaired mitochondrial gene transcription and function [23]. Impaired mitophagy and accelerated apoptosis provoked by GSDI intermediary metabolites also appear to exacerbate mitochondrial dysfunction [24, 25, 26, 27]. Among 14 patients with GSDIa, urinary and plasma samples demonstrated higher levels of TCA cycle intermediates and plasma acylcarnitines, suggesting mitochondrial decompensation [28, 29]. Interestingly, this type of cumulative mitochondrial dysfunction appears to promote tumorigenic pathways and the development of hepatocellular adenomas and carcinomas, well‐known long‐term complications of GSDIa, in *G6PC* knockout mice [30]. Taken together, metabolic derangements inherent to GSDIa appear to cause progressive mitochondrial dysfunction over time and increase the risk for GSDIa‐related complications, including fulminant metabolic crisis. The apparent cumulative nature of mitochondrial dysfunction observed in GSDIa may help explain why patients do not pass away in early infancy but face a continued risk of mortality throughout their lifespan. However, this hypothesis does not fully explain the presentation of our youngest patient (patient 2) who had adequate metabolic control prior to her demise.

Existing clinical practice guidelines for GSDIa recommend routine metabolic lab and image screening, around the clock cornstarch administration, dietary modifications, and medication to address secondary disease manifestations [1]. While there have been updates to the management of GSDIa over the past 20 years, the cornerstone of management remains cornstarch therapy [31]. Additionally, our cases occurred between 2007 and 2022, allowing for evaluation of these events along with updated treatment strategies like continuous glucose monitoring and extended‐release cornstarch [3]. As three of our cases with presumed metabolic control appear to suggest, existing GSDI screening labs may misclassify some patients as having adequate metabolic control because mitochondrial health is not being assessed. In fact, there is a growing body of literature that suggests we need to place a greater emphasis on long‐term mitochondrial health in GSDI [6]. Incorporating measurements of mitochondrial function into current surveillance strategies may allow for the introduction of targeted mitochondrial therapeutics to prevent progressive mitochondrial injury. Surveillance could be achieved through commercially available mitochondrial respiratory chain enzymatic assays and cellular oxygen consumption rate, though invasive tissue sampling limits their current widespread clinical utility [32, 33, 34]. Less specific plasma screening, including pyruvate‐lactate ratio, beta hydroxybutyrate:acetatoacetate ratio, glutathione measurements, white blood cell CoQ10 levels, plasma amino acids, and urine organic acids, could serve as proxy measurements; however, if identified, GSDIa patients with increased mitochondrial dysfunction could be treated with readily accessible antioxidant therapy, including thiamine, vitamin E, coenzyme Q10, and N‐acetylcysteine, among others [11, 15, 35]. More research is needed to assess the feasibility of this approach in GSDI and how this may affect morbidity and mortality.

In summary, we present five cases of acute metabolic decompensation in GSDIa characterized by refractory lactic acidosis despite hyper‐ or euglycemia. While we may never determine what ultimately caused these patients' demise, we propose that progressive secondary mitochondrial dysfunction may play a role as recent literature suggests a close interaction between glycogen and mitochondrial metabolism. Establishing routine surveillance of mitochondrial function in GSDIa patients may facilitate initiation of targeted antioxidant therapy to mitigate progressive mitochondrial dysfunction, alleviate chronic symptom burden, and potentially help prevent fulminant metabolic crises in these individuals. Further research is needed to test these hypotheses.

## Author Contributions

All authors reviewed the manuscript and contributed to the intellectual content presented. H.G. reviewed patient data and wrote the manuscript. N.S. contributed to patient data collection and reviewed the manuscript, L.M. contributed to patient data collection and reviewed the manuscript, V.S. contributed to patient data collection and reviewed the manuscript, R.G. contributed to the discussion, reviewed and edited the manuscript, D.D.D.L. conceptualized the work, contributed to patient data collection, and reviewed and edited the manuscript. All authors approved submission of this article for publication.

## Funding

The authors have nothing to report.

## Ethics Statement

The authors have nothing to report.

## Consent

The authors have nothing to report.

## Conflicts of Interest

D.D.D.L. has received consulting fees from Zealand Pharma, Rezolute, Rhythm Pharmaceuticals, Confo Therapeutics, Amidebio, Spruce Therapeutics, Ligand Pharmaceuticals, Ultragenyx Pharmaceutical, Fortress Biotech, and Twist Pharmaceuticals for work not related to this manuscript. D.D.D.L. has received research funding/contracts from Zealand Pharma, Hanmi Pharmaceuticals, Twist Pharmaceuticals, Rezolute, Rhythm Pharmaceuticals, Moderna, and Ultragenyx for work not related to this manuscript. R.G. has received consulting fees from Minovia Therapeutics, research funding/contracts from Saol Therapeutics, and equipment support from Aligent, outside the submitted work. The remaining authors declare nothing to disclose.

## Supporting information


**Table S1:** Available lab profiles of Glycogen Storage Disease Type Ia patients at time of presentation with fulminant metabolic crisis.

## Data Availability

Data sharing not applicable to this article as no datasets were generated or analysed during the current study.
