# Supplementary material for: Retrospective Case Series of Fulminant Metabolic Crisis in GSDIA: Persistent Lactic Acidosis Despite Correction of Hypoglycemia May Reflect Secondary Mitochondrial Dysfunction
Source: JIMD Rep. 2025 Dec 25;67(1):e70059. doi: 10.1002/jmd2.70059 (PMC12741212; doi:10.1002/jmd2.70059)
Supplement: Supplementary file 1 — Table S1: Available lab profiles of Glycogen Storage Disease Type Ia patients at time of presentation with fulminant metabolic crisis. [file JMD2-67-e70059-s001.docx]

**Supplementary Table 1.** Available lab profiles of Glycogen Storage Disease Type Ia patients at time of presentation with fulminant metabolic crisis.

| Case | Infectious | Cardio/Renal | GI | Endocrine/Metabolic | Autopsy findings |
| --- | --- | --- | --- | --- | --- |
| 1 | Blood culture: no growth.  Urine culture: no growth. Peritoneal culture: no WBCs, no organisms seen; no growth.  Vaginal culture: negative for N. gonorrhoeae.  No evidence of acute appendicitis from amputated appendix.  WBC 17.6 K/uL (RR: 4.5-13.5)  Plts 351 K/uL (150-400) | BUN 10 mg/dL (RR: 7-18)  Cr 2.5 mg/dL (0.4-1.0) | ALT 149 U/L  (RR: 5-30)  AST 545 U/L (10-30)  Lipase 2064 U/L (25-110)  GGT 237 U/L  (12-22) | PG 140 mg/dL (RR: 72-135)  Urine ketones 3.9 mmol/L (negative)  pH 6.80  HCO3 <5 mmol/L (20-26)  Uric acid 12.5 mg/dL (2.6-6.0) | Not performed |
| 2^*^ | Blood culture: no growth.  Urine culture: no growth.  Respiratory culture: moderate WBCs, rare gram-positive cocci; normal colonizing flora of corynebacterium sp.  Viral PCR panel: adenovirus, RSV, Flu A/B, Parainfluenza 1/2/3, human metapneumovirus, rhinovirus all negative.  WBC 27.2 K/uL (6-17)  Plts 683 K/uL (150-400) | BUN 31 mg/dL (5-17)  Cr 1.9 mg/dL (0.1-0.4)  ECHO: tricuspid valve regurgitation, trivial mitral valve regurgitation, left ventricular systolic shortening is qualitatively hyperdynamic, no aortic valve regurgitation, no pericardial effusion. | ALT 36 U/L  (5-45)  AST 140 U/L (20-60)  GGT 87 U/L  (5-16)  Lipase 33U/L  (15-130) | TSH 1.30 mIU/mL (0.5-3.8)  fT4 0.7 ng/dL (1.0-1.8)  T3 <0.4 ng/mL (0.8-2.2)  PG 87 mg/dL (67-121)  pH 6.93  HCO3 <5 mmol/L (21-27)  Lactate 24.1 mmol/L (0.5-1.6) | Hepatomegaly, mild nephromegaly, diffuse bronchopneumonia with diffuse alveolar damage, brain swelling. Bacterial and fungal cultures from the lung, spleen and blood negative. Viral studies from lung negative. |
| 3^*^ | Blood culture: no growth.  Urine culture: no growth.  WBC 44.8 K/uL (4.5-13.5)  Plts 236 K/uL (150-400) | BUN 32 mg/dL (5-17)  Cr 3.4 mg/dL (0.2-0.5)  Troponin 0.83 ng/mL (<0.3)  ECHO: normal left and right ventricular size and qualitatively normal systolic shortening. | ALT 1321 U/L  (10-35)  AST 3746 U/L  (15-40)  GGT 32 U/L  (11-21)  Lipase 282 U/L  (25-120) | PG 19 mg/dL (70-106)  Small urine ketones (negative)  pH 6.709  HCO3 4.5 mmol/L (20-26)  Lactate >27 mmol/L (0.5-1.6)  Ammonia 251.6 umol/L (9-33)  CK 3043 U/L (60-365) | Bilateral bronchopneumonia with patchy pulmonary hemorrhage. Bacterial and fungal cultures negative from lung and spleen. |
| 4 | WBC 49.5 CMM  (4.8-10.8)  Plts 490 CMM (130-400)  Blood culture: no growth.  Urine culture: no growth.  MRSA/VRE PCR negative. | BUN 19 mg/dL (5-26)  Cr 2.76 mg/dL (0.5-1.5)  Troponin <0.03 ng/mL (<0.06) | ALT 65 IU/L  (2-38)  AST 190 IU/L  (9-37)  Lipase 265 U/L  (22-51) | PG 509 mg/dL  Trace urine ketones (negative)  HbA1c 4.9% (4.9-6.0)  HCO3 <5 mEq/L (24-31)  Lactate >20 mEq/L (0.5-2.2) | Not performed |
| 5+ | Blood culture: no growth.  Respiratory culture: many WBCs; no organisms seen.  Urine culture: no growth.  Mycoplasma pneumoniae PCR: negative  Mycoplasma pneumoniae AB, IgM 0.11 U/L  (<=0.76 U/L)  Rapid Flu A/B, COVID-19 & RSV: negative  Viral PCR panel: VZV, EBV, human parechovirus, HHV-6, CMV, parvovirus B-19, enterovirus, adenovirus all negative.  ESR 27 mm/hr (0-20)  CRP 3.4 mg/dL (0-0.9) | BUN 14 mg/dL (7-18)  Cr 1.33 mg/dL (0.3-0.8)  BNP 218.3 pg/mL (0-100)  Troponin 0.03 ng/mL  ECHO: moderate secundum atrial septal defect with bidirectional flow (L>R). Trivial tricuspid valve regurgitation. Mild interventricular septal flattening in systole. Hyperdynamic left ventricular systolic shortening. Normal right ventricular systolic shortening. | ALT 79 U/L  (10-35)  AST 313 U/L (15-45)  Lipase 47 U/L  (23-300) | PG 239 mg/dL (60-115)  Negative urine ketones (negative)  pH 6.927  HCO3 2.3 mmol/L (22-26)  Lactate >25 mmol/L (0.5-2.2)  Lactate-pyruvate ratio 30  (10-20)  Urine 2OH-isovaleric acid 357  mg/g Cr  2keto-glutaric acid 32  mg/g Cr  Plasma alloisoleucine 10.4 umol/L (<=0.0)  Plasma alanine 193.1 umol/L  (89-440)  Plasma proline 116.8 umol/L  (83-346)  Plasma C4OH carnitine was noted (0.22 umol/L) | Acute pancreatitis with areas of necrosis. Bacterial and fungal cultures of spleen with no growth. |

*Siblings; ^+^Presented labs are from time of fulminant metabolic crisis as opposed to initial hospital presentation based on rapid change in lactate level for this patient. Initial lab work at time of hospital presentation is detailed in the text. Abbreviations: GI (gastrointestinal), WBCs (white blood cells), K/uL (thousands per microliter), RR (reference range), Plts (platelets), BUN (blood urea nitrogen), Cr (creatinine), ALT (alanine aminotransferase), AST (aspartate aminotransferase), GGT (gamma-glutamyl transpeptidase), PG (plasma glucose), HCO3 (bicarbonate), RSV (respiratory syncytial virus), flu (influenza), ECHO (echocardiogram), TSH (thyroid stimulating hormone), fT4 (free thyroxine), T3 (triiodothyronine), CK (creatine kinase), CMM (cells per cubic millimeter), MRSA (methicillin-resistant staphylococcus aureus), VRE (vancomycin-resistant enterococci), PCR (polymerase chain reaction), HbA1c (hemoglobin A1c), AB (antibody), VZV (varicella zoster virus), EBV (Epstein-Barr virus), HHV-6 (human herpesvirus 6), CMV (cytomegalovirus), ESR (erythrocyte sedimentation rate), CRP (c-reactive protein), BNP (B-type natriuretic peptide), L (left), R (right).
